# Metformin-Induced Nightmares: An Uncommon Event

**DOI:** 10.7759/cureus.64251

**Published:** 2024-07-10

**Authors:** Bosky Modi, Devi P Suravajjala, James Case, Priya Velumani

**Affiliations:** 1 Internal Medicine, Texas Tech University Health Sciences Center, Odessa, USA; 2 Endocrinology, Diabetes and Metabolism, Texas Tech University Health Sciences Center, Odessa, USA

**Keywords:** adverse effect, rare, nightmares, type 2 diabetes mellitus, metformin

## Abstract

Metformin is well-known in the treatment of type 2 diabetes mellitus (DM). Metformin has become a drug of choice due to its affordability, cost-effectiveness, and established safety record. It primarily works by inhibiting hepatic gluconeogenesis. Common side effects include gastrointestinal issues, with rare complications, such as lactic acidosis and vitamin B12 malabsorption. This study discussed a 72-year-old male with type 2 DM who experienced recurrent nightmares upon initiating metformin, which ceased after discontinuation. The mechanism of metformin-associated nightmares remains poorly understood. Despite metformin's benefits, this case highlights the importance of recognizing rare adverse effects like nightmares, which can significantly impact patients' quality of life.

## Introduction

Metformin, a biguanide, stands often as the initial drug of choice for treating type 2 diabetes mellitus (DM) [1]. Its roots in herbal medicine trace back to the 1950s, underpinning its global usage and effectiveness [2]. Metformin's popularity stems from its effectiveness, affordability, weight neutrality, and safety [3]. Metformin has additional benefits of reducing inflammation and lowering cardiovascular risks, seemingly unrelated to its glucose-lowering properties [3]. While metformin exhibits diverse effects on glucose metabolism, its primary action in lowering blood sugar in type 2 DM patients stems from its inhibition of hepatic gluconeogenesis [4]. Metformin's primary side effects include gastrointestinal symptoms such as diarrhea, nausea, and abdominal discomfort. However, a rare but severe complication is lactic acidosis [3]. Metformin also causes vitamin B12 malabsorption, as evidenced by numerous case reports and studies [5]. Although rare, nightmares are sporadically reported as a side effect in patients using metformin [6]. Changes in blood glucose levels, influenced by metformin initiation, may impact sleep patterns, potentially contributing to metformin-induced nightmares [7,8].

## Case presentation

A 72-year-old male presents to our endocrinology clinic for the management of type 2 DM. His medical history was notable for hypertension, dyslipidemia, presbycusis, depression, chronic back pain, and obstructive sleep apnea (OSA). He had no relevant family history. He denied smoking, alcohol, or illicit drug use. His home medications included sitagliptin, dapagliflozin, semaglutide, metoprolol, venlafaxine, lisinopril, hydrochlorothiazide, and simvastatin with a recent addition of metformin due to uncontrolled hemoglobin A1c. Vital signs were within normal range (Table 1). All laboratory values were within normal range except hemoglobin A1c (Table 2). Physical examination was unremarkable.

**Table 1:** Vitals signs on presentation.

Vital signs	Value	Reference values
Blood pressure	123/77 mmHg	<120/80 mmHg
Heart rate	80 beats/min	60-100 beats/min
Oxygen saturation	98% on room air	95-100%
Body temperature	36.2°C	36.5°C-37.3°C
Respiratory rate	20 breaths/min	12-20 breaths/min

**Table 2:** Laboratory values on presentation. AST: aspartate aminotransferase; ALT: alanine transaminase

Labs	Value	Reference values
White blood cells	5.9 k/μL	4.5-11.0 k/μL
Hemoglobin	15.4 g/dL	13.8-17.2 g/dL
Hematocrit	43.5%	40.7-50.3%
Platelet count	239 k/μL	150-400 k/μL
Chloride	98 mEq/L	96-106 mEq/L
Bicarbonate	27 mEq/L	22-29 mEq/L
Sodium	136 mEq/L	135-145 mEq/L
Potassium	4.6 mEq/L	3.5-5.0 mEq/L
Total bilirubin	0.7 mEq/L	0.1-1.2 mg/dL
Alkaline phosphatase	82 U/L	44-147 U/L
AST	19 U/L	10-40 U/L
ALT	21 U/L	7-56 U/L
Calcium	9.6 mg/dL	8.6-10.2 mg/dL
Total protein	9.1 g/dL	6.0-8.3 g/dL
Albumin	4.7 g/dL	3.5-5.0 g/dL
Hemoglobin A1c	7.8%	4-5.6%

At the three-month follow-up visit, the hemoglobin A1c remained the same. However, he reported recurrent nightmares for the past two weeks. He recalled experiencing similar symptoms when initially prescribed metformin in 2001, with the disappearance of nightmares after discontinuation. Due to the provided history, we discontinued his metformin and added glimepiride. We continued the rest of his medications. On a follow-up visit after a month, he reported a complete resolution of his nightmares.

## Discussion

DM is a widespread disease in the United States affecting 37.3 million people or 11.3% of the US population [9]. Metformin, a cornerstone medication in the management of type 2 DM, is frequently prescribed, particularly for overweight individuals with normal kidney function [10]. Its therapeutic benefits extend beyond effective glycemic control, as it aids in reducing hyperinsulinemia, facilitating weight loss, improving lipid profiles, wide security margin, and enhancing endothelial function in patients with type 2 DM [10,11].

Despite its efficacy and widespread use, metformin is not without its potential side effects. While generally well-tolerated, it can lead to adverse reactions such as lactic acidosis and common gastrointestinal disturbances, including nausea, vomiting, stomach upset, and diarrhea [12]. Additionally, although rare, metformin may precipitate hypoglycemia, mainly when used in conjunction with other antidiabetic medications [10].

One notable side effect associated with metformin, albeit uncommon, is the occurrence of nightmares, characterized by intensely dysphoric dreams, typically manifest during the latter stages of sleep, particularly during the rapid eye movement (REM) stage of sleep [13,14]. They are often linked to heightened psychological distress and may even precede the onset of conditions such as post-traumatic stress disorder (PTSD) or psychotic experiences [15]. Various medications can precipitate nightmares as an adverse effect such as antihypertensive agents such as beta-blockers, angiotensin-converting enzyme inhibitors, calcium channel blockers; statins like atorvastatin; antiepileptic drugs like valproic acid, lamotrigine; antimicrobial like erythromycin; antidepressants like fluoxetine; and antipsychotics agent like chlorpromazine [16]. Pharmacological agents impacting neurotransmitters like norepinephrine, serotonin, and dopamine are strongly linked to patient-reported nightmares [17].

The etiology of drug-induced nightmares, including those potentially triggered by metformin, remains multifactorial and lacks clear pharmacologic explanations [18]. The change in blood sugar levels after starting metformin therapy might be the only plausible reason for the occurrence of nightmares [19]. Nightmares might stem from nocturnal hypoglycemia. Yet, metformin monotherapy typically doesn't induce hypoglycemia, especially without factors like fasting, malnutrition, intense exercise, or advanced age [20]. The cerebral blood glucose level has an effect on the body's mechanism for dreaming [18]. Despite the rarity of metformin-induced nightmares, it's essential to recognize and address this potential side effect, particularly given the prevalence of sleep disturbances among individuals with type 2 DM [18-20].

In our case, the patient took multiple medications that could precipitate nightmares, such as statins, antidepressants, and beta blockers. However, the onset of nightmares following the recent introduction of metformin, without any adjustments to his ongoing medications, suggested that metformin was the likely cause. Rather than discontinuing all medicines at once, step-by-step discontinuation and rechallenge appear to be the most efficient way to confirm rare adverse effects from medications. Given his previous history of metformin-associated nightmares, his recurrence of nightmares after restarting metformin, followed by resolution on discontinuation, serves as a positive rechallenge test confirming this rare adverse effect that remains underrecognized worldwide.

## Conclusions

Metformin is a commonly prescribed oral medication for lowering glucose in type 2 DM. Despite metformin's effectiveness, affordability, and safety in managing type 2 DM, it causes various side effects from the commonly known gastrointestinal disturbances to the rare occurrence of life-threatening lactic acidosis. However, metformin-induced nightmares remain a rare and often underrecognized adverse effect. Its mechanism leading to nightmares needs to be better understood. However, cessation of metformin leads to complete resolution of symptoms within weeks to months. Recognizing and addressing rare side effects like nightmares from metformin can significantly improve the quality of life for patients, ensuring the therapeutic benefits of the medication remain uncompromised by adverse reactions.

## References

[1] Vieira IH, Barros LM, Baptista CF, Rodrigues DM, Paiva IM (2022). Recommendations for practical use of metformin, a central pharmacological therapy in type 2 diabetes. Clin Diabetes.

[2] Flory J, Lipska K (2019). Metformin in 2019. JAMA.

[3] Sanchez-Rangel E, Inzucchi SE (2017). Metformin: clinical use in type 2 diabetes. Diabetologia.

[4] LaMoia TE, Shulman GI (2021). Cellular and molecular mechanisms of metformin action. Endocr Rev.

[5] de Jager J, Kooy A, Lehert P (2010). Long term treatment with metformin in patients with type 2 diabetes and risk of vitamin B-12 deficiency: randomised placebo controlled trial. Br Med J.

[6] Wiwanitkit S, Wiwanitkit V (2012). Metformin and sleep disorders. Indian J Endocrinol Metab.

[7] Nakajima H, Kaneita Y, Yokoyama E (2008). Association between sleep duration and hemoglobin A1c level. Sleep Med.

[8] Heiss WD, Pawlik G, Herholz K, Wagner R, Wienhard K (1985). Regional cerebral glucose metabolism in man during wakefulness, sleep, and dreaming. Brain Res.

[9] (2022). National diabetes statistics report: estimates of diabetes and its burden in the United States. https://stacks.cdc.gov/view/cdc/85309.

[10] Nasri H, Rafieian-Kopaei M (2014). Metformin: current knowledge. J Res Med Sci.

[11] Dutta S, Shah RB, Singhal S, Dutta SB, Bansal S, Sinha S, Haque M (2023). Metformin: a review of potential mechanism and therapeutic utility beyond diabetes. Drug Des Devel Ther.

[12] Scheen AJ, Paquot N (2013). Metformin revisited: a critical review of the benefit-risk balance in at-risk patients with type 2 diabetes. Diabetes Metab.

[13] Rek S, Sheaves B, Freeman D (2017). Nightmares in the general population: identifying potential causal factors. Soc Psychiatry Psychiatr Epidemiol.

[14] Gieselmann A, Ait Aoudia M, Carr M (2019). Aetiology and treatment of nightmare disorder: State of the art and future perspectives. J Sleep Res.

[15] Semiz UB, Basoglu C, Ebrinc S, Cetin M (2008). Nightmare disorder, dream anxiety, and subjective sleep quality in patients with borderline personality disorder. Psychiatry Clin Neurosci.

[16] Agrawal PK, Gautam A, Pursnani N, Parihar A, Singh B (2021). An unusual side effect of metformin - nightmare and abnormal dreams. J Diabetol.

[17] Pagel JF, Helfter P (2003). Drug induced nightmares - an etiology based review. Hum Psychopharmacol.

[18] Thompson DF, Pierce DR (1999). Drug-induced nightmares. Ann Pharmacother.

[19] Voloshyna D, Sandhu QI, Khan S (2022). An unusual association between metformin and nightmares: a case report. Cureus.

[20] Yanto TA, Huang I, Kosasih FN, Lugito NP (2018). Nightmare and abnormal dreams: rare side effects of metformin. Case Rep Endocrinol.

